# Area of center of pressure in closed eye setting as a measure of postural sway: Association with frailty and functional capacity in older adults with diabetes

**DOI:** 10.1371/journal.pone.0333608

**Published:** 2025-10-09

**Authors:** Remi Kodera, Yoshiaki Tamura, Yuji Murao, Fumino Yorikawa, Ai Iizuka, Kazuhito Oba, Kenji Toyoshima, Yuko Chiba, Joji Ishikawa, Atsushi Araki

**Affiliations:** 1 Department of Diabetes, Metabolism, and Endocrinology, Tokyo Metropolitan Institute for Geriatrics and Gerontology, Tokyo, Japan; 2 Center for Comprehensive Care and Research for Prefrailty, Tokyo Metropolitan Institute for Geriatrics and Gerontology, Tokyo, Japan; 3 Department of Cardiology, Tokyo Metropolitan Institute for Geriatrics and Gerontology, Tokyo, Japan; National Trauma Research Institute, AUSTRALIA

## Abstract

Older adults with diabetes mellitus are at a higher risk of frailty, which can lead to disability and death; therefore, effective frailty screening is necessary in such populations. However, evidence linking sway meter indices to frailty or functional capacity remains limited. This cross-sectional study aimed to investigate the association of postural stability, assessed using a sway meter, with frailty and functional capacity in older patients with and without diabetes. Data from 362 older outpatients (149 with diabetes and 213 without) who visited the Frailty Clinic between 2021 and 2022 were analyzed. The Kihon Check List was used to define frailty and the Short Physical Performance Battery to assess functional capacity. The sway meter indices included locus length (Lo) and area of the center of pressure (Ao) with open eyes, locus length (Lc) and area of center of pressure (Ac) with closed eyes, and the Romberg ratio (Ac/Ao). Univariate and multivariate analyses were performed to determine associations. All sway meter indices were higher in patients with diabetes. In this group, Lo, Ao, and Ac levels were significantly higher in those with frailty, while Lo, Lc, Ao, and Ac levels were higher in those with low functional capacity. Receiver operating characteristic analyses showed that Ao and Ac had relatively high area under the curve for both diagnoses. Binominal logistic regression analyses revealed that Ac was significantly associated with frailty in patients with diabetes after adjusting for age, sex, HbA1c, cognitive function, number of medications, and several diabetic complication indices, including loss of Achilles tendon reflexes (odds ratio, 1.107; 95% confidence interval, 1.001–1.225; p = 0.048). Ac was also significantly associated with low functional capacity. These findings suggest that the area of the center of pressure, especially in a closed-eye setting, is associated with frailty and functional capacity in older adults with diabetes.

## Introduction

As life expectancy increases and populations age worldwide, the number of older adults with diabetes is increasing. In fact, the number of patients aged ≥ 65 years with diabetes is expected to reach 276 million by 2045 [1].

Frailty is a state in which a person becomes vulnerable to external stress during aging, with diabetes mellitus being a strong risk factor. Since patients with diabetes and frailty are susceptible to functional disability, mortality, and lower quality of life [2], early diagnosis and intervention are vital.

A sway meter detects spatiotemporal changes in the body. Various types of sway meters, such as plate type and, more recently, wearable, have been developed. For the plate type, the participant stands on the sway meter with eyes open or closed, and the center of pressure movement parameters at each position are recorded [3].

Postural sway, assessed using a sway meter, is associated with the risk of falls in older adults [4,5]. Mahoney et al. showed that trunk sway, detected using a wearable device worn on the lower back, can predict falls in community-dwelling older adults [4]. However, data on the association between sway meter indices and frailty or functional capacity are limited. The length, area, and mean speed of sway, assessed using a plate-type sway meter, are associated with several types of frailty statuses.

These statuses are determined using the Cardiovascular Health Study (CHS) criteria, Clinical Frailty Scale, and Frailty Index based on the deficit accumulation model (FI) in hospitalized older adults [6]. However, the studies were not performed in outpatient clinics for chronic diseases, and no data are available for patients with diabetes.

Postural sway is greater in patients with diabetes than in those without diabetes [7]. Thus, postural control in patients with diabetes may be important for preventing frailty and improving functional capacity. Diabetic peripheral neuropathy is a risk factor for postural instability [8], but other factors may be involved. Although the effects of other sensory functions than neuropathy, including visual and vestibular function, arterial stiffness, and polypharmacy, on sway in patients with diabetes [9] have been investigated, the evidence of the influence of these factors on postural sway remains lacking. Also, it remains unclear which sway meter parameters are associated with frailty and functional capacity in patients with diabetes.

In this study, we aimed to determine which the sway meter parameters are most strongly associated with frailty and functional capacity in older patients with diabetes. Frailty was assessed using The Kihon Checklist (KCL) [10], a screening tool based on the Comprehensive Geriatric Assessment (CGA), while functional capacity was evaluated using the Short Physical Performance Battery (SPPB) [11].

## Methods

### Participants

The data of patients aged ≥ 65 years who visited our Frailty Clinic between January 1, 2021, and December 31, 2022, were evaluated. The clinic was established to provide a comprehensive assessment of frailty, sarcopenia, cognition, depression, nutrition, medications, and social status in patients with cardiometabolic diseases. These diseases include diabetes mellitus, hypertension, or dyslipidemia, with symptoms of frailty such as fatigue and slow gait speed [12]. Diabetes mellitus was diagnosed based on the physician’s electronic medical records. A history of angina pectoris, myocardial infarction, symptomatic cerebral infarction, or bleeding was defined as cardiovascular disease (CVD). In total, 362 patients (149 with diabetes and 213 without) who underwent balance testing using a sway meter were included. Patients with a history of advanced cancer, severe acute diseases, conditions requiring hospitalization, or severely impaired activities of daily living and/or cognitive function were excluded from the study. Those who could not stand on the sway meter for at least 30 s were also excluded.

### Assessment of frailty status and functional capacity

We used the KCL as an indicator of frailty, as it is based on a comprehensive geriatric assessment and has been validated and utilized in Japan as a predictive tool for long-term care needs [10,13,14]. Additionally, compared with the CHS criteria, which comprise only five items, the KCL is more suitable for evaluating temporal changes in frailty after an intervention for frailty prevention.

It comprises 25 questions based on the CGA, including those on instrumental activities of daily living, physical function, nutrition, oral function, cognition, depressive mood, and social withdrawal. Patients who test positive for ≥ 8 items are diagnosed with frailty. The validity of the KCL has been demonstrated in China [15], Thailand [16], Brazil [17], Spain [18], and Italy [19]. The KCL has also shown the highest predictive ability for frailty (as defined by the CHS criteria) among three self-administered questionnaires [20].

Functional ability is assessed using the SPPB, which includes three tests: balance, walking speed, and five-time repeated chair rise tests. Patients scoring ≤ 9 out of a total of 12 are classified as having low functional capacity [11].

### Evaluation of postural stability

Postural stability while standing was evaluated using a sway meter CP-5000 (Anima, Tokyo, Japan). The patients were instructed to stand for 30 s with their feet shoulder-width apart. The test was repeated twice—once with eyes open and once with eyes closed. The following postural sway indices were assessed according to the manufacturer’s instructions: locus length with open (Lo) and closed (Lc) eyes; area of the center of pressure with open (Ao) and closed (Ac) eyes; the Romberg ratio (R; Ac divided by Ao). A plate-type sway meter identical to the one used in this study has good reliability (coefficient: 0.89–0.95) [21].

### Other assessments

Patient height, weight, body mass index, and blood pressure were measured on the day of the frailty evaluation. Blood test data under ad lib settings were also used for the analyses. These included the levels of glycated hemoglobin A1c (HbA_1c_), serum albumin, low-density lipoprotein cholesterol, high-density lipoprotein cholesterol, triglycerides, creatinine, cystatin C, and the estimated glomerular filtration rate.

The Achilles tendon reflex (ATR) was tested using a Babinski reflex hammer. If the reflex was absent or significantly attenuated on either side, it was considered a loss of ATR. Subjective visual impairment was recorded as positive if the patient answered “Yes” to the question, “Do you have difficulty in seeing?”.

Sarcopenia was determined using the Asian Working Group for Sarcopenia 2019 criteria [22]. Cognitive function was evaluated using the Mini-Mental State Examination (MMSE) [23]. The types of medications (oral medicines) were counted using copied prescriptions. Nutritional state was evaluated using the Mini Nutritional Assessment Short-Form [24], and depressive mood was evaluated with the Geriatric Depression Scale [25]. The number of medications was defined as the number of oral drug types. All other clinical information was obtained from the medical records.

### Ethics approval statement

Written informed consent was obtained from all the participants. This study was conducted in accordance with the Declaration of Helsinki, and the study protocol was approved by the Ethics Committee of Tokyo Metropolitan Geriatric Hospital (R15-20).

### Statistical analysis

We first compared clinical parameters and postural sway indices by diabetes status using the Mann–Whitney *U* test and chi-square test. We calculated the required number of patients with diabetes. We estimated that when treating Ac as a dichotomous variable, the prevalence of KCL frailty in the high Ac group with diabetes was 0.5, whereas the prevalence in the low Ac group with diabetes was 0.25 (OR = 2); set α = 0.05 and power (1 − β) = 0.8. Based on these parameters, the required sample size was determined as 116 using ClinCalc (https://clincalc.com/stats/samplesize.aspx, accessed July 2025).

In patients with diabetes, we compared postural sway indices by frailty or functional capacity using the Mann–Whitney *U* test.

Correlations between the two numerical variables were assessed using Spearman’s rank correlation coefficient. Next, receiver operating characteristic (ROC) analyses were performed, and the area under the curve (AUC) was calculated to identify the parameter that best predicts the risk of frailty and low functional capacity in patients with diabetes. Cutoff values for determining frailty and low functional capacity were identified using the Youden Index. Finally, binomial logistic regression analysis was conducted in patients with diabetes, with frailty or low functional capacity as the objective variable, and each sway meter index as the explanatory variable adjusted for covariates. We selected covariates known to affect the prevalence of frailty, such as HbA1c [26], cognitive function [27], polypharmacy [28], diabetic complications [29], and CVD. Three models were tested: Model 1, adjusted for age, sex, and loss of ATR; Model 2, further adjusted for HbA1c, MMSE scores, and number of medications; Model 3, included all variables in Model 1, plus subjective visual impairment, cystatin C-based estimated glomerular filtration rate (eGFR-cysC), and history of CVD; Model 4, Model 2 plus duration of diabetes and use of insulin or sulfonylurea (SU). We also added another model, Model 2’, in the logistic analysis where Ac was a dichotomous variable in Model 2. High Ac was defined as Ac ≥ 4.30 cm^2^ and Ac ≥ 4.34 cm^2^, the cutoff values derived from ROC curve analyses for KCL-defined frailty and functional impairment, respectively. The data were accessed for research purposes on 21/10/2024. All statistical analyses were performed using SPSS ver. 26 (IBM Corp., Armonk, NY, USA), and statistical significance was set at p < 0.05.

## Results

### Clinical characteristics and postural sway indices of participants

The clinical characteristics of the study participants are listed in Table 1. The median age was 81 years. The prevalence of frailty and low functional capacity in patients with diabetes was 39.2% and 35.8%, respectively, compared with 37.6% and 33.5% in patients without diabetes. In patients with diabetes, the median HbA1c level was 7.2%, median disease duration 16 years, and the proportion of patients with ATR loss was 48.5%. All sway indices were higher in patients with diabetes than in those without.

**Table 1:** Clinical characteristics of study participants.

	Total (n = 362)	Patients with diabetes (n = 149)	Patients without diabetes (n = 213)	p
Age	81[77–85]	81 [77–84]	81[77–86]	0.281
Females (%)	63.5	62.4	64.3	0.712
Body mass index (kg/m^2^)	23.7[21.1–25.8]	24.6 [22.3–26.6]	23.0[20.4–25.3]	<0.001
Systolic blood pressure (mmHg)	138[124–150]	138 [124–151]	137[124–150]	0.622
Diastolic blood pressure (mmHg)	75[66–82]	73 [65–83]	75[67–82]	0.168
Albumin (g/dl)	4.2[4.0–4.4]	4.2 [4.0–4.4]	4.2[4.0–4.4]	0.589
Hemoglobin (g/dl)	13.0[12.0–14.0]	12.9 [12.0–13.8]	13.0[12.0–14.2]	0.559
HbA1c (%)	6.51[5.9–7.1]	7.2 [6.7–7.6]	6.0[5.7–6.2]	<0.001
GA (%)	16.8[14.5–18.3]	19.1[16.7–21.2]	15.0[13.7–15.8]	<0.001
Triglyceride (mg/dl)	131[85–156]	145 [94–174]	122[80–149]	0.004
LDL-C (mg/dl)	104[84–123]	99 [78–119]	107[88–125]	0.016
HDL-C (mg/dl)	58[47–67]	56 [44–63]	60[50–69]	0.004
Creatinine (mg/dl)	1.01[0.71–1.13]	1.06 [0.69–1.17]	0.97[0.72–1.12]	0.890
eGFR-cysC (ml/min/1.73m^2^)	60.0[44.9–74.3]	60.2 [45.8–73.7]	59.8[42.8–75.4]	0.985
MMSE	27[26 –30 ]	27 [26 –30 ]	27[26 –30 ]	0.454
MoCA-J	21[18 –24]	21 [18 –24 ]	21[18 –24 ]	0.610
Number of Medications	6[3 –8]	7[5 –9]	5[3 –7 ]	<0.001
Statin (%)	64.4	69.8	60.6	0.071
Insulin or SU (%)		14.1		
disease duration of diabetes (years)		16[9.5–25]		
ATR loss (%)	24.9	48.5	4.1	<0.001
visual impairment (%)	44.4	40.8	46.9	0.250
CVD (%)	22.3	28.8	17.8	0.015
Frailty (%)	38.2	39.2	37.6	0.754
Lower functional capacity (%)	34.4	35.8	33.5	0.649
MNA-SF	12[11 –14 ]	12[11 –14 ]	12[10 –14]	0.217
GDS-15	4[2 –6]	4[2 –6]	4[2 –6]	0.945
Lo(cm)	60.0[41.1–73.1]	64.1[46.1–80.5]	57.1[38.7–67.7]	0.001
Lc(cm)	82.4[51.2–102.5]	90.1[59.0–114.1]	77.0[47.2–90.8]	<0.001
Ao(cm^2^)	3.0[1.5–3.5]	3.4[1.6–4.0]	2.8[1.5–3.2]	0.034
Ac(cm^2^)	4.3[1.8–5.1]	5.0[2.2–6.2]	3.8[1.6–4.6]	0.001
Romberg ratio	1.5[0.9–1.9]	1.7[1.0–2.0]	1.4[0.9–1.8]	0.054

median [25–75% tile].

LDL-C: low-density lipoprotein cholesterol, HDL-C: high-density lipoprotein cholesterol, eGFR-cysC: cystatin C based estimated glomerular filtration rate, GA: glycoalbumin, MMSE: mini-mental state examination, MoCA-J: Japanese version of Montreal Cognitive Assessment, SU: sulfonylurea, ATR: Achilles tendon reflex, CVD: cardiovascular disease, MNA-SF: Mini Nutritional Assessment – Short Form, GDS-15: Geriatric Depression Scale – 15, Lo: moving length with open eyes, Lc: moving length with closed eyes, Ao: moving area with open eyes, Ac: moving area with closed eyes.

### Postural sway indices in patients with diabetes by frailty or low functional capacity

Postural sway indices in patients with diabetes based on frailty or low functional capacity are shown in Table 2. Patients with frailty had significantly higher Lo (p = 0.044), Ao (p = 0.001), and Ac (p = 0.005) values than those without frailty, whereas there were no significant differences in Lc and R (Table 2). Likewise, Lo (p = 0.017), Lc (p = 0.048), Ao (p = 0.005), and Ac (p = 0.016) values were higher in patients with low functional capacity than in those with high functional capacity (Table 3).

**Table 2:** Postural sway indices in older patients with diabetes by the frailty based on KCL.

	Frailty (+) (n = 58)	Frailty (-) (n = 90)	p
Lo(cm)	**69.4[47.0–88.1]**	**60.9[44.1–75.5]**	**0.044**
Lc(cm)	100.7[59.7–132.3]	83.9[58.4–99.9]	0.081
Ao(cm^2^)	**4.0[2.1–4.6]**	**3.0[1.4–3.3]**	**0.001**
Ac(cm^2^)	**6.3[2.9–7.8]**	**4.2[1.9–4.9]**	**0.005**
Romberg ratio	1.7[1.0–2.2]	1.6[1.0–1.9]	0.978

KCL: Kihon Checklist, SPPB: Short Physical Performance Battery, Lo: moving length with open eyes, Lc: moving length with closed eyes, Ao: moving area with open eyes, Ac: moving area with closed eyes.

**Table 3:** Postural sway indices in older patients with diabetes by the low functional capacity based on SPPB.

	Low functional capacity (+) (n = 53)	Low functional capacity (-) (n = 95)	P
Lo(cm)	**72.7[48.5-91.6]**	**59.5[45.6-72.0]**	**0.017**
Lc(cm)	**101.7[62.8-132.4]**	**84.4[57.0-105.5]**	**0.048**
Ao(cm^2^)	**4.4[1.7-5.0]**	**2.8[1.5-3.3]**	**0.005**
Ac(cm^2^)	**7.0[2.5-9.9]**	**4.0[2.1-5.1]**	**0.016**
Romberg ratio	1.7[1.0-2.1]	1.6[1.0-1.9]	0.776

KCL: Kihon Checklist, SPPB: Short Physical Performance Battery, Lo: moving length with open eyes, Lc: moving length with closed eyes, Ao: moving area with open eyes, Ac: moving area with closed eyes.

### ROC analyses of sway meter indices to discriminate frailty or low functional capacity

**Fig 1:**
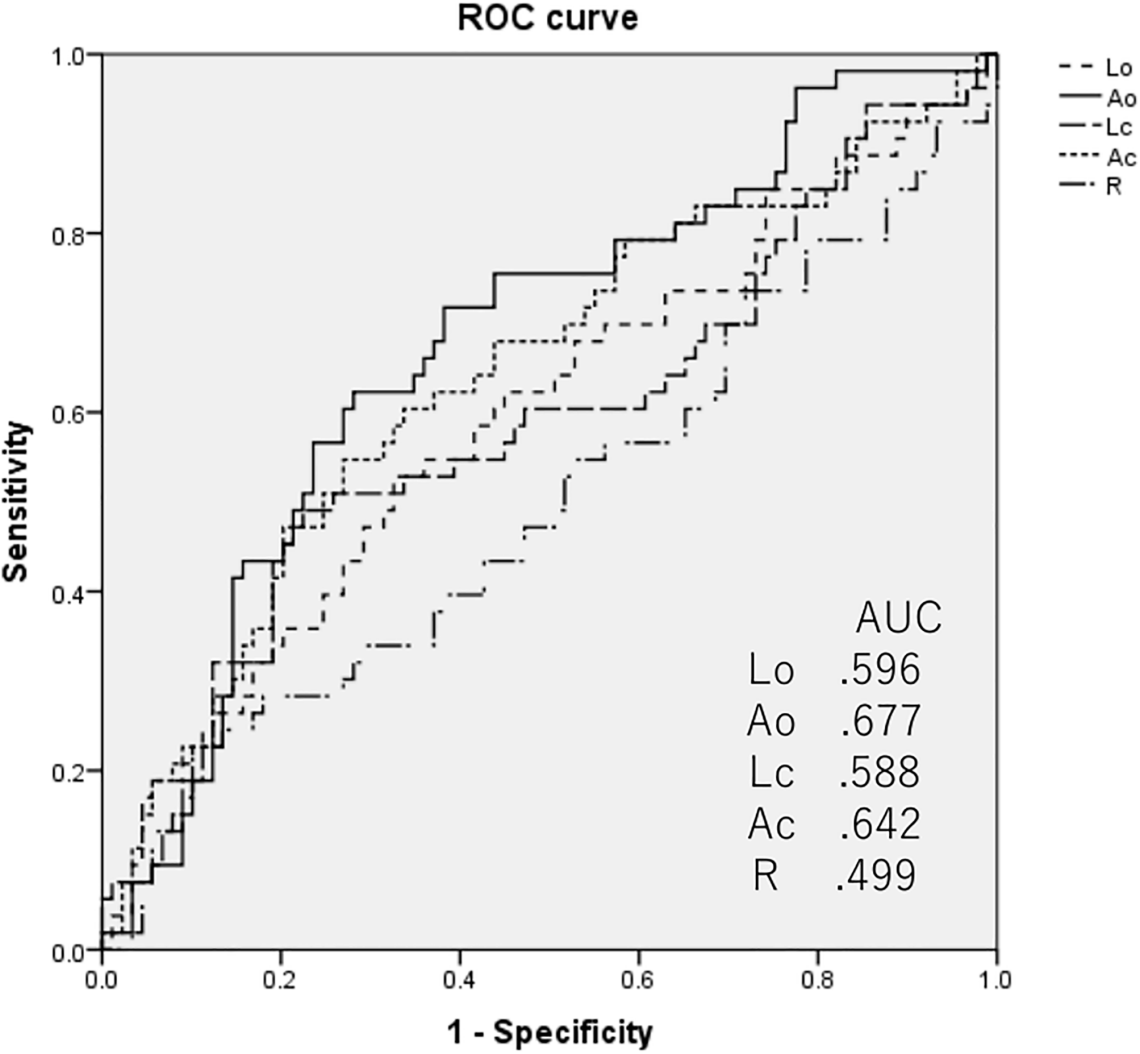
ROC analyses of the indices of the sway meter to determine their ability to distinguish frailty by KCL.

**Fig 2:**
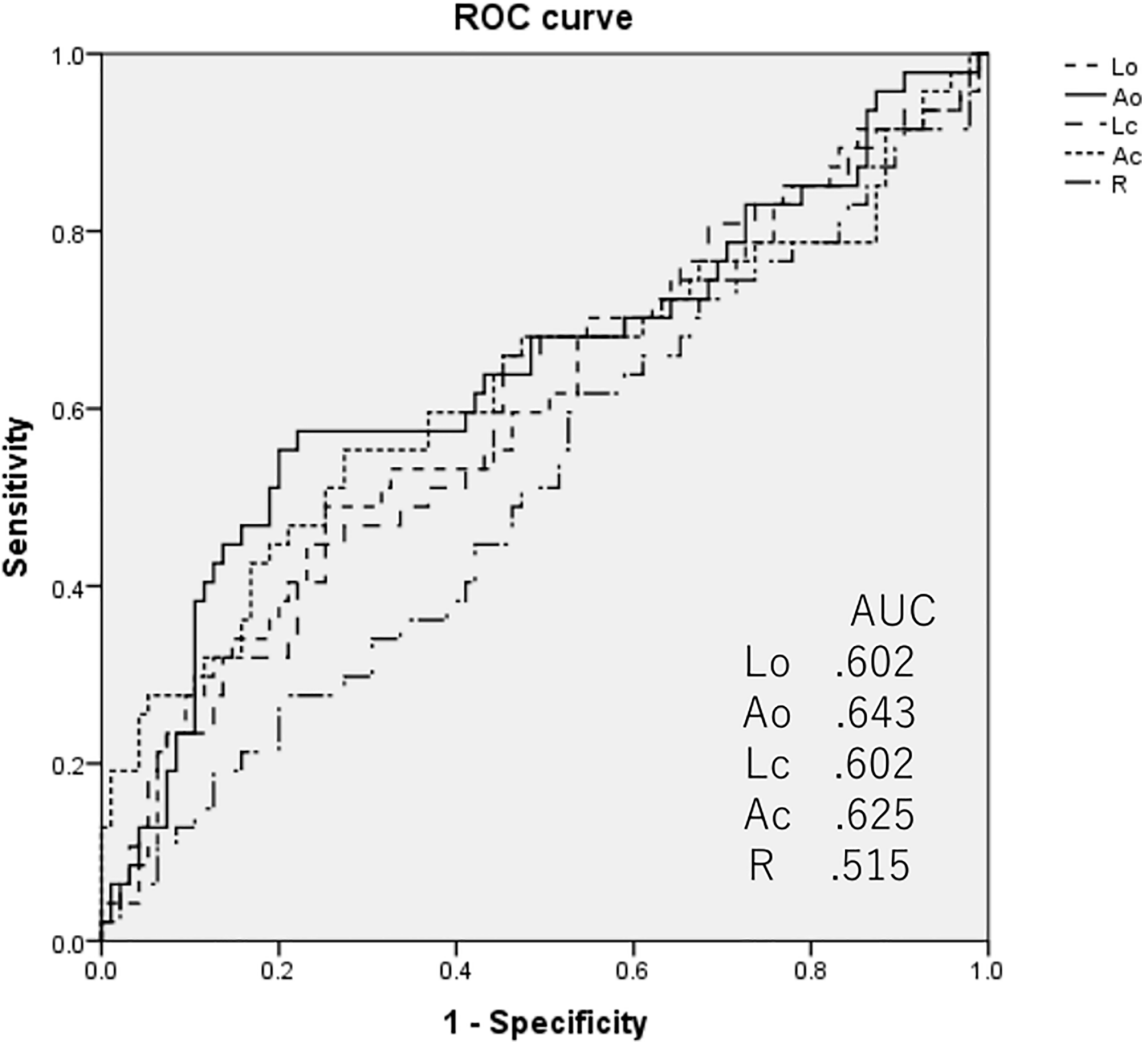
ROC analyses of the indices of the sway meter to determine their ability to distinguish low functional capacity by SPPB.

The results of the ROC analysis are shown in Fig 1 and 2. The AUC values of Ao and Ac for discriminating frailty were the first and second highest at 0.677 and 0.642, respectively (Fig 1). The AUC of Ao and Ac for discriminating low functional capacity were also relatively higher than those of the other indices, with values of 0.643 and 0.625 (Fig 2). Thus, we selected these two indices for further analysis. The cutoff values of Ao and Ac were 3.03 and 4.30 cm^2^ for KCL frailty and 3.45 and 4.34 cm^2^ for low functional capacity, respectively.

### Binomial logistic regression analysis to assess the associations of Ac and Ao with frailty or low functional capacity

Table 4 presents the results of binomial logistic regression analyses targeting frailty and low functional capacity in patients with diabetes. We found significant associations between Ac and frailty in Model 1 (odds ratio [OR], 1.105 per 1 cm^2^ increase; 95% confidence interval [CI], 1.004–1.217; p = 0.041) and Model 2 (OR, 1.107 per 1 cm^2^ increase; 95% CI, 1.001–1.225; p = 0.048). In this model, the MMSE score and number of medications were significantly associated with the prevalence of frailty (p = 0.044 and p = 0.023, respectively). The association between Ac and frailty remained significant in Model 3 (OR, 1.121 per 1 cm^2^ increase; 95% CI, 1.002–1.254; p = 0.047) (Table 4). However, the association between Ao and frailty was not significant in the multivariate analyses (S1 Table).

**Table 4:** Binominal logistic regression analysis for the association between moving area with closed eyes (Ac) and KCL-defined frailty in older patients with diabetes.

	Model 1		Model 2		Model 3	
	OR (95%CI)	p	OR (95%CI)	p	OR (95%CI)	p
Ac	**1.105(1.004–1.217)**	**0.041**	**1.107(1.001–1.225)**	**0.048**	**1.121(1.002–1.254)**	**0.047**
Age	**1.099(1.020–1.184)**	**0.013**	1.060(0.976–1.151)	0.168	1.063(0.969–1.165)	0.196
Sex (Males)	0.978(0.436–2.195)	0.957	0.856(0.351–2.090)	0.733	0.921(0.326–2.610)	0.876
Loss of ATR	0.555(0.251–1.225)	0.145	0.435(0.184–1.025)	0.057	0.455(0.152–1.360)	0.159
HbA1c			1.144(0.635–2.060)	0.654		
MMSE			**0.841(0.710–0.995)**	**0.044**		
Number of Medications			**1.165(1.022–1.328)**	**0.023**		
visual impairment					1.938(0.721–5.209)	0.190
eGFR-cysC					**0.970(0.942–0.999)**	**0.042**
CVD					1.827(0.584–5.718)	0.301

Model 1: Adjusted for age, sex and loss of ATR

Model 2: Adjusted for age, sex, loss of ATR, HbA1c, MMSE, and number of medications

Model 3: Adjusted for age, sex, loss of ATR, visual impairment, eGFR-CysC, and CVD

*Ac: moving area with closed eyes, ATR: Achilles tendon reflex, MMSE: Mini-mental state examination, CVD: cardiovascular disease

Similarly, Ac was positively associated with low functional capacity in Model 1 (OR, 1.172 per 1 cm^2^ increase; 95% CI, 1.054–1.304; p = 0.003), Model 2 (OR, 1.172; 95% CI, 1.053–1.304; p = 0.004) and Model 3 ((OR, 1.189 per 1 cm^2^ increase; 95% CI, 1.044–1.354; p = 0.009) (Table 5). The association between Ao and low functional capacity was not significant in multivariate analyses (S2 Table).

**Table 5:** Binominal logistic regression analysis for the association between Ac and SPPB-defined low functional capacity in older patients with diabetes.

	Model 1		Model 2		Model 3	
	OR (95%CI)	p	OR (95%CI)	P	OR (95%CI)	p
Ac	**1.172(1.054–1.304)**	**0.003**	**1.172(1.053–1.304)**	**0.004**	**1.189(1.044–1.354)**	**0.009**
Age	1.033(0.958–1.115)	0.400	1.034(0.951–1.124)	0.437	1.048(0.949–1.159)	0.353
Sex (Males)	**0.331(0.128–0.858)**	**0.023**	**0.305(0.113–0.821)**	**0.019**	**0.240(0.066–0.874)**	**0.030**
Loss of ATR	**3.283(1.396–7.719)**	**0.006**	**2.942(1.233–7.024)**	**0.015**	**5.044(1.510–16.845)**	**0.009**
HbA1c			0.928(0.506–1.701)	0.809		
MMSE			0.994(0.849–1.164)	0.942		
Number of Medications			1.043(0.920–1.184)	0.508		
visual impairment					2.087(0.667–6.524)	0.206
eGFR-cysC					0.986(0.954–1.018)	0.382
CVD					2.227(0.667–7.434)	0.193

Model 1: Adjusted for age, sex and loss of ATR

Model 2: Adjusted for age, sex, loss of ATR, HbA1c, MMSE, and number of medications

Model 3: Adjusted for age, sex, loss of ATR, visual impairment, eGFR-CysC, and CVD

*Ac: moving area with closed eyes, ATR: Achilles tendon reflex, MMSE: Mini-mental state examination, CVD: cardiovascular disease

In the analysis where the duration of diabetes and use of insulin or SU were further added to Model 2 (Model 4), the significant association between Ac and frailty and low functional capacity persisted (S3 Table and S4).

In Model 2’, where Ac was treated as a dichotomous variable, the results were similar to those in Model 2. The association between Ac and frailty and low functional capacity was similarly observed (S5 Table and S6). The OR of high Ac for frailty was 2.661, and that for low functional capacity was 3.005.

## Discussion

In this study, we evaluated sway in those with DM and without and investigated the association between sway indices in DM patients.

All sway indices were higher in patients with diabetes than in those without, consistent with previous findings [7].

To the best of our knowledge, this is the first study to show a positive association between Ac, sway meters indices, and the prevalence of frailty and low functional capacity in older outpatients with diabetes. The association was robust and persisted after adjusting for age, sex, neuropathy, HbA1c level, cognitive function, polypharmacy, visual impairment, renal function, and history of CVD.

The similarity in results for both frailty and low functional capacity, as assessed using the SPPB, was consistent with those of a previous report, which showed that a low SPPB score is a useful screening tool for detecting frailty [30,31].

Our findings suggest that impaired postural stability in patients with diabetes, frailty, or functional capacity manifests as loss of visual information. Postural instability increases in patients with diabetes in the absence of visual information [32,33]. One possible explanation for this phenomenon is peripheral neuropathy. Even in non-diabetic older adults, in two cohort studies from the United States (National Health and Nutrition Examination Survey and The Atherosclerosis Risk in Communities Study), the prevalence of peripheral neuropathy, as defined by monofilament testing, was 25.4% and 31.2%, respectively, among individuals aged ≥ 70 years [34]. In older patients with diabetes, diabetic peripheral neuropathy (DPN) is frequently present [35]. DPN may impair postural balance in patients with diabetes mellitus, and ATR loss is a major clinical sign of DPN. Patients with DPN frequently experience impaired deep sensations such as vibration [36]. In our multivariate analysis, ATR loss was linked with frailty and significantly associated with low functional capacity; however, Ac was associated with frailty and functional capacity independent of ATR. This suggests that sway meter indices in closed eye settings may reflect postural instability due to causes other than DPN.

Alternatively, vestibular dysfunction can cause postural instability in the absence of visual information. Patients with diabetes, especially those with long disease duration, are likely to show vestibular dysfunction, which can be observed in those without DPN [37]. Although the mechanism of vestibular dysfunction remains unclear, brain small vessel diseases or white matter lesions may be involved in abnormalities in postural control and balance. In the Rotterdam Scan Study, nearly all adults aged ≥ 80 years had some white matter lesions; the percentages of individuals completely free of subcortical and periventricular white matter lesions were 0% and 5%, respectively [38]. Therefore, white matter integrity may be crucial for the efficient transfer of visual, proprioceptive, and vestibular feedback in the brain. Altered white matter integrity in the frontal or occipital forceps, detected using diffusion tensor imaging on brain MRI, is associated with disturbances in balance and postural control in older adults [39]. However, since vestibular function is not routinely evaluated in clinical settings, no studies have directly compared the timing of onset between vestibular dysfunction and DPN. Further studies are warranted to clarify the temporal relationship and clinical significance and assess the relationship between abnormal cerebral white matter integrity and postural control in patients with diabetes.

In our study, the association between Ac and frailty remained significant after adjusting for HbA1c levels, MMSE scores, and the number of medications.

Hyperglycemia has been reported to be associated with a higher incidence of functional disability [26]. However, we found no association between HbA1c levels and frailty. This may be because HbA1c levels in our participants were substantially well controlled (75% tile: 6.7–7.6%, Table 1), and small differences in HbA1c might not have influenced the prevalence of frailty. Zaslavsky et al. showed that lower glycemic levels (mean glucose level ≤150 mg/dl or HbA1c ≤ 6.9%) and hyperglycemia were associated with incident frailty [40]. This could be attributed to the higher incidence of hypoglycemia in patients with low HbA1c levels. Although we had no data on the frequency of hypoglycemia, the proportion of patients who used insulin or SU was low (14.1%). Moreover, the association between Ac and frailty, as well as low functional capacity, remained significant in Model 4 after further adjusting for the use of insulin or SU and duration of diabetes (S3 Table and S4). It is unlikely that high Ac was induced by hypoglycemia.

Cognitive impairment may be associated with impaired balance in patients with diabetes. Previous reports have shown that impairments in certain cognitive domains, particularly executive function, are associated with falls and gait disturbances in patients with diabetes [27,41]. In this study, the MMSE score, which includes executive function [42], also showed an association with the prevalence of frailty in multivariate analysis (Table 4). However, since the association of Ac with frailty did not change after adjusting for cognitive function, it is unlikely that cognitive function affected postural instability in patients with diabetes.

The number of medications administered was significantly associated with frailty. Polypharmacy can lead to frailty through multimorbidity, adverse drug events such as hypoglycemia, and poor adherence. This may also explain the association between postural instability and frailty, as some reports have directly linked polypharmacy to postural instability [28]. However, our multivariate analysis showed that sway meter indices could predict the presence of frailty independent of polypharmacy.

Multivariate analysis showed that visual impairment, eGFR-cysC, and a history of CVD did not affect the association between Ac (a sway meter index) and frailty or low functional capacity. This suggests that the association is independent of other diabetic complications, such as retinopathy, nephropathy, and CVD. The renal function of the patients in our study was not significantly impaired and was far higher than the level at which it could affect the increased prevalence of frailty [43].

The strength of this study lies in its simultaneous evaluation of several indices of the sway meter, frailty status, functional capacity, and various covariates, including blood samples, in older outpatients with diabetes. The results of this study revealed the association between the most useful index of the sway meter and low functional capacity and frailty based on the CGA.

However, this study has some limitations. First, owing to its cross-sectional design, the causal relationship between sway indices and frailty/functional capacity was unclear. Second, DPN and visual impairment were assessed using only ATR and subjective visual impairment tests, which may have led to inaccuracies in the diagnoses of DPN and retinopathy. Finally, this study was conducted at a single institution in Japan and limited to patients who presented with relatively good glucose control and fewer diabetic complications at the frailty clinic. Therefore, these findings should be interpreted with caution when applied to the general population of older patients with diabetes. Longitudinal studies with a large sample of diabetic populations are necessary to clarify whether abnormalities in sway meter indices are associated with the incidence of frailty or low functional capacity.

Regardless of the study’s limitations, we have identified an association between Ac and frailty (based on the CGA) and low functional capacity in older outpatients with diabetes. Since these associations are independent of DPN, glycemic control, cognitive impairment, polypharmacy, and other diabetic complications, assessing postural sway in older adults with diabetes may be important for predicting the presence of frailty and low functional capacity. Further studies are needed to clarify the underlying mechanisms of these associations. Additionally, research should determine whether intervention with balance training in older patients with diabetes—especially those with abnormal sway indices—could help prevent deterioration in frailty or functional capacity.

## Supporting information

S1 Table Binominal logistic regression analysis for the association between moving area with open eyes (Ao) and Kihon Checklist-defined frailty in older patients with diabetes. (DOCX)

S2 Table Binominal logistic regression analysis for the association between Ao and Short Physical Performance Battery-defined low functional capacity in older patients with diabetes. (DOCX)

S3 Table Binominal logistic regression analysis for the association between moving area with closed eyes (Ac) and KCL-defined frailty in older patients with diabetes where plus duration of diabetes and use of insulin or SU were further added on Model 2 (Model 4). (DOCX)

S4 Table Binominal logistic regression analysis for the association between Ac and SPPB-defined low functional capacity in older patients with diabetes where plus duration of diabetes and use of insulin or SU were further added on Model 2 (Model 4). (DOCX)

S5 Table Binominal logistic regression analysis for the association between moving area with closed eyes (Ac) and KCL-defined frailty in older patients with diabetes where Ac was treated as dichotomous variables. (DOCX)

S6 Table Binominal logistic regression analysis for the association between Ac and SPPB-defined low functional capacity in older patients with diabetes where Ac was treated as dichotomous variables. (DOCX)
