# Supplementary material for: Area of center of pressure in closed eye setting as a measure of postural sway: Association with frailty and functional capacity in older adults with diabetes
Source: PLoS One. 2025 Oct 9;20(10):e0333608. doi: 10.1371/journal.pone.0333608 (PMC12510599; doi:10.1371/journal.pone.0333608)
Supplement: S1 Table — (DOCX) [file pone.0333608.s001.docx]

**Supplementary Table 1A. Binominal logistic regression analysis for the association between moving area with open eyes (Ao) and KCL-defined frailty in older patients with diabetes**

|  | **Model 1** | | **Model 2** | | **Model 3** | |
| --- | --- | --- | --- | --- | --- | --- |
|  | **OR (95%CI)** | **p** | **OR (95%CI)** | **p** | **OR (95%CI)** | **p** |
| Ao | 1.054(0,907-1.225) | 0.494 | 1.013(0.862-1.190) | 0.875 | 1.030(0,863-1.230) | 0.743 |
| Age | **1.102(1.024-1.186)** | **0.010** | 1.069(0.986-1.160) | 0.104 | 1.076(0.982-1.178) | 0.115 |
| Sex (Men) | 0.974(0.454-2.088) | 0.945 | 0.891(0.391-2.033) | 0.784 | 0.961(0.366-2.523) | 0.936 |
| Loss of ATR | 0.644(0.303-1.372) | 0.254 | 0.580(0.261-1.289) | 0.181 | 0.690(0.256-1.854) | 0.461 |
| HbA1c |  |  | 1.186(0.682-2.063) | 0.545 |  |  |
| MMSE |  |  | 0.866(0.739-1.016) | 0.077 |  |  |
| Number of Medications |  |  | **1.130(1.002-1.275)** | **0.046** |  |  |
| visual impairment |  |  |  |  | 2.541(0.988-6.532) | 0.053 |
| eGFR-cysC |  |  |  |  | **0.974(0.948-1.000)** | **0.047** |
| CVD |  |  |  |  | 1.897(0.651-5.531) | 0.241 |

Model 1: Adjusted for age, sex and loss of ATR

Model 2: Adjusted for age, sex, loss of ATR, HbA1c, MMSE, and number of medications

Model 3: Adjusted for age, sex, loss of ATR, visual impairment, eGFR-CysC, and CVD

＊Ao: moving area with open eyes, ATR: Achilles tendon reflex, MMSE: Mini-mental state examination, CVD: cardiovascular disease
