# Supplementary material for: Area of center of pressure in closed eye setting as a measure of postural sway: Association with frailty and functional capacity in older adults with diabetes
Source: PLoS One. 2025 Oct 9;20(10):e0333608. doi: 10.1371/journal.pone.0333608 (PMC12510599; doi:10.1371/journal.pone.0333608)
Supplement: S2 Table — (DOCX) [file pone.0333608.s002.docx]

**Supplementary Table 2. Binominal logistic regression analysis for the association between Ao and SPPB-defined low functional capacity in older patients with diabetes**

|  | **Model 1** | | **Model 2** | | **Model 3** | |
| --- | --- | --- | --- | --- | --- | --- |
|  | **OR (95%CI)** | **p** | **OR (95%CI)** | **P** | **OR (95%CI)** | **p** |
| Ao | 1.162(0.982-1.375) | 0.081 | 1.156(0.973-1.375) | 0.100 | 1.208(0.997-1.464) | 0.053 |
| Age | 1.045(0.971-1.124) | 0.241 | 1.040(0.960-1.128) | 0.337 | 1.056(0.962-1.158) | 0.252 |
| Sex (Men) | 0.454(0.195-1.056) | 0.067 | 0.423(0.176-1.015) | 0.054 | 0.338(0.110-1.036) | 0.058 |
| Loss of ATR | **3.298(1.474-7.379)** | **0.004** | **3.055(1.347-6.930)** | **0.008** | **5.662(1.826-17.554)** | **0.003** |
| HbA1c |  |  | 0.992(0.838-1.143) | 0.979 |  |  |
| MMSE |  |  | 0.979(0.838-1.143) | 0.784 |  |  |
| Number of Medications |  |  | 1.034(0.922-1.161) | 0.566 |  |  |
| visual impairment |  |  |  |  | 2.700(0.917-7.952) | 0.072 |
| eGFR-cysC |  |  |  |  | 0.983(0.955-1.012) | 0.251 |
| CVD |  |  |  |  | 2.421(0.777-7.544) | 0.127 |

Model 1: Adjusted for age, sex and loss of ATR

Model 2: Adjusted for age, sex, loss of ATR, HbA1c, MMSE, and number of medications

Model 3: Adjusted for age, sex, loss of ATR, visual impairment, eGFR-CysC, and CVD

＊Ao: moving area with open eyes, ATR: Achilles tendon reflex, MMSE: Mini-mental state examination, CVD: cardiovascular disease
