# Supplementary material for: Area of center of pressure in closed eye setting as a measure of postural sway: Association with frailty and functional capacity in older adults with diabetes
Source: PLoS One. 2025 Oct 9;20(10):e0333608. doi: 10.1371/journal.pone.0333608 (PMC12510599; doi:10.1371/journal.pone.0333608)
Supplement: S3 Table — (DOCX) [file pone.0333608.s003.docx]

**Supplementary Table 3. Binominal logistic regression analysis for the association between moving area with closed eyes (Ac) and KCL-defined frailty in older patients with diabetes　 where plus duration of diabetes and use of insulin or SU were further added on Model 2 (Model 4).**

|  | **Model 4** | |
| --- | --- | --- |
|  | **OR (95%CI)** | **p** |
| Ac | **1.131(1.016-1.258)** | **0.024** |
| Age | 1.046(0.956-1.144) | 0.324 |
| Sex (Men) | 0.990(0.367-2.666) | 0.984 |
| Loss of ATR | 0.414(0.147-1.166) | 0.095 |
| Duration of diabetes | 1.002(0.955-1.051) | 0.947 |
| Use of insulin or SU | 1.195(0.292-4.889) | 0.805 |
| HbA1c | 1.228(0.635-2.373) | 0.541 |
| MMSE | 0.863(0.719-1.035) | 0.112 |
| Number of Medications | 1.118(0.964-1.296) | 0.142 |

Model 4: Adjusted for age, sex, loss of ATR, duration of diabetes, antidiabetic drugs, HbA1c, MMSE, and number of medications

＊Ac: moving area with closed eyes, ATR: Achilles tendon reflex, SU: sulfonylurea, MMSE: Mini-mental state examination, CVD: cardiovascular disease
