# Supplementary material for: Area of center of pressure in closed eye setting as a measure of postural sway: Association with frailty and functional capacity in older adults with diabetes
Source: PLoS One. 2025 Oct 9;20(10):e0333608. doi: 10.1371/journal.pone.0333608 (PMC12510599; doi:10.1371/journal.pone.0333608)
Supplement: S4 Table — (DOCX) [file pone.0333608.s004.docx]

**Supplementary Table 4. Binominal logistic regression analysis for the association between Ac and SPPB-defined low functional capacity in older patients with diabetes where plus duration of diabetes and use of insulin or SU were further added on Model 2 (Model 4).**

|  | **Model 4** | |
| --- | --- | --- |
|  | **OR (95%CI)** | **P** |
| Ac | **1.195(1.060-1.347)** | **0.004** |
| Age | 1.056(0.961-1.160) | 0.260 |
| Sex (Men) | 0.364(0.121-1.096) | 0.072 |
| Loss of ATR | 2.318(0.816-6.586) | 0.115 |
| Duration of diabetes | 0.971(0.920-1.024) | 0.279 |
| Use of insulin or SU | **4.567(1.036-20.124)** | **0.045** |
| HbA1c | 0.947(0.466-1.924) | 0.881 |
| MMSE | 0.981(0.818-1.176) | 0.834 |
| Number of Medications | 1.044(0.894-1.219) | 0.587 |

Model 4: Adjusted for age, sex, loss of ATR, duration of diabetes, antidiabetic drugs, HbA1c, MMSE, and number of medications

＊Ac: moving area with closed eyes, ATR: Achilles tendon reflex, SU: sulfonylurea, MMSE: Mini-mental state examination, CVD: cardiovascular disease
