# Supplementary material for: Area of center of pressure in closed eye setting as a measure of postural sway: Association with frailty and functional capacity in older adults with diabetes
Source: PLoS One. 2025 Oct 9;20(10):e0333608. doi: 10.1371/journal.pone.0333608 (PMC12510599; doi:10.1371/journal.pone.0333608)
Supplement: S5 Table — (DOCX) [file pone.0333608.s005.docx]

**Supplementary Table 5. Binominal logistic regression analysis for the association between moving area with closed eyes (Ac) and KCL-defined frailty in older patients with diabetes** **where Ac was treated as dichotomous variables.**

|  | **Model 2’** | |
| --- | --- | --- |
|  | **OR (95%CI)** | **p** |
| High Ac | **2.661(1.110-6.378)** | **0.028** |
| Age | 1.073(0.986-1.168) | 0.101 |
| Sex (Men) | 0.890(0.367-2.158) | 0.796 |
| Loss of ATR | 0.444(0.189-1.042) | 0.062 |
| HbA1c | 1.109(0.605-2.036) | 0.738 |
| MMSE | 0.867(0.730-1.029) | 0.103 |
| Number of Medications | **1.157(1.015-1.319)** | **0.029** |

Model 2’: Adjusted for age, sex, loss of ATR, HbA1c, MMSE, and number of medications

Ac: moving area with closed eyes, ATR: Achilles tendon reflex, MMSE: Mini-mental state examination, CVD: cardiovascular disease

⋆High Ac was defined as Ac ≥ 4.30 cm^2^, the cutoff values derived from ROC curve analyses for KCL-defined frailty.
