# Supplementary material for: Area of center of pressure in closed eye setting as a measure of postural sway: Association with frailty and functional capacity in older adults with diabetes
Source: PLoS One. 2025 Oct 9;20(10):e0333608. doi: 10.1371/journal.pone.0333608 (PMC12510599; doi:10.1371/journal.pone.0333608)
Supplement: S6 Table — (DOCX) [file pone.0333608.s006.docx]

**Supplementary Table 6. Binominal logistic regression analysis for the association between Ac and SPPB-defined low functional capacity in older patients with diabetes where Ac was treated as dichotomous variables.**

|  | **Model 2’** | |
| --- | --- | --- |
|  | **OR (95%CI)** | **P** |
| High Ac | **3.005(1.237-7.298)** | **0.015** |
| Age | 1.041(0.958-1.131) | 0.340 |
| Sex (Men) | **0.387(0.153-0.977)** | **0.045** |
| Loss of ATR | **2.917(1.251-6.799)** | **0.013** |
| HbA1c | 0.959(0.530-1.736) | 0.891 |
| MMSE | 1.025(0.873-1.204) | 0.763 |
| Number of Medications | 1.037(0.914-1.176) | 0.574 |

Model 2’: Adjusted for age, sex, loss of ATR, HbA1c, MMSE, and number of medications

Ac: moving area with closed eyes, ATR: Achilles tendon reflex, MMSE: Mini-mental state examination, CVD: cardiovascular disease

⋆High Ac was defined as Ac ≥ 4.34 cm^2^, the cutoff values derived from ROC curve analyses for SPPB-defined low functional capacity.
